# Antimicrobial, antidiabetic, antiviral, and antioxidant activities of fucoidan extracted from the brown seaweed *Padina pavonica*

**DOI:** 10.1186/s12896-025-01004-1

**Published:** 2025-07-09

**Authors:** Faiza M. A. Akl, Mostafa M. El-Sheekh, Mofida E. M. Makhlof, Suzan I. Ahmed

**Affiliations:** 1 Biological and Geological Sciences Department, Faculty of Education, Alexandria University, Alexandria, Egypt; 2 Botany Department, Faculty of Science, Tanta University, Tanta, 31527 Egypt; 3 Botany and Microbiology Department, Faculty of Science, Damanhour University, Damanhour, Egypt

**Keywords:** Seaweed, Fucoidan, Extraction, *Padina pavonica*, Brown algae, Antimicrobial, Antidiabetic

## Abstract

Macroalgae are considered a promising biological source of active substances, and sulfated polysaccharides are among the most important compounds with potential applications. Here, fucoidan from *Padina pavonica* collected from the Rocky Bay of Abu Qir in Alexandria was analyzed for its ash, water, protein, sulfated groups, elemental content, total sugars, and uronic acid levels. Additionally, its monosaccharides were qualitatively and quantitatively determined using high-performance liquid chromatography for fucose detection, which is the main fingerprint of fucoidans. The isolated fucoidan was characterized using FTIR, scanning electron microscopy, EDX, and thermogravimetric analysis (TGA). Its biological activities, including antiviral, antioxidant, antimicrobial, and antidiabetic effects, were then evaluated. The yields of fucoidan, fucose, and sulfate were found to be 17.8 ± 0.23%, 34.45%, and 9.52 ± 0.19%, respectively. It inhibited HSV-1 with an inhibition percentage of 30.89 ± 0.84. The maximum Ferric Reducing Antioxidant Power (FRAP) value reached 81.82 ± 1.44% at 1000 µg/ml. *Padina* fucoidan (PF) showed the largest inhibition zone of 18 mm against Methicillin-Resistant *Staphylococcus aureus* (MARSA) (ATCC 4330) with an MIC of 1.25 mg/L. It also demonstrated a promising inhibitory effect on α-Amylase enzyme, reaching 75.69 ± 1.05 at a concentration of 1000 µg/ml. We conclude that *Padina pavonica* is an excellent producer of fucoidan, with a significant sulfate content that enhances its biological activities, especially its antidiabetic properties.

## Introduction

Our forefathers have long utilized Mother Nature as a remarkable supply of valuable natural compounds for a variety of purposes, such as biological, cosmetic, therapeutic, and nutritional [1], one of these valuable natural compounds is sulfated polysaccharides (SP), which comprise dozens of families. They exist in living organisms and mainly have many promising biological applications, such as antiviral activity, as SP and their derivatives have been shown in numerous studies to have strong inhibitory effects against a wide range of viruses [2]. SP can be produced chemically or naturally and contains sulfate moieties in its carbohydrate backbone. They occur in the cell walls of seaweeds and are extremely rare in mangrove plants, so studying them is essential for drug discovery initiatives [2].

According to their backbone, fucoidan is a general word for a polysaccharide with fucose as a footprint [3]. Brown seaweed cell walls and several marine invertebrates, including sea cucumbers [4] and sea urchins [5], contain fucoidans, which can make up as much as 20% of the dry weight of brown algae cell walls. Due to their ability to combat inflammation, viruses, blood clotting, cancers, and thrombosis, brown seaweed polysaccharides, such as fucoidan, are focused on extensive biological effectiveness studies [6]. These fucose-rich sulfated polysaccharides have garnered a lot of interest lately because of their demonstrated bioactivities, which include antibacterial, adhesive, anticancer, anti-inflammatory, and anticoagulant qualities [7].

Brown seaweeds can be used to extract fucoidans, commonly with hot water or acid treatment methods, using a temperature range from 70 to 100 °C. The next step is to precipitate alginate using CaCl_2_ to separate pure fucoidans. Finally, precipitation in alcohol is used to separate the fucoidans [8]. Alginate, laminarin, and phenolic chemicals in the fucoidans can be eliminated by further purifying procedures after extractions. The extraction process should prevent significant depolymerization and sulfate loss with the obtaining of pure fucoidan, as its activities are linked to their purity, molecular weight, composition, and sulfation pattern, even though details regarding the precise structural components that cause them are so far unknown. Subcritical water extraction [9], microwave-assisted extraction [10], ultrasound-assisted extraction [11], and enzyme-assisted extraction [12] are examples of extraction techniques that have been developed to increase the quantity and quality of fucoidans.

Herein, fucoidan was extracted from the alga *P. pavonica* (PF) using the hot water extract method, then purified and characterized using many parameters like HPLC, EDX, FTIR, and TGA and then tested for the first time for its antidiabetic via testing its inhibition effect upon α-Amylase enzyme for its antioxidant activity using FRAP assay for its antiviral activity and its antimicrobial activity.

## Materials and methods

### *Padina pavonica* collection

*Padina pavonica* was collected in October 2024. After being completely cleaned with seawater and distilled water, *P. pavonica* was air-dried in the shade, then oven-dried at 60 °C before being ground into a powder and stored in firmly sealed sheets for future research. Taxonomic identification was done with the aid of [13] and confirmed through the Algae Base website’s identification and habitat data [14].

### Extraction of PF

The extraction method of Yang et al. [15] was used to extract PF from powdered masses. In short, 20 g of powder was extracted 2 times at 65 °C by stirring for two hours using 400 mL of distilled water. After combining the extracted materials, ten minutes of centrifugation take place at 13,000 × g. To precipitate alginic acid, 1% CaCl_2_ was used; after that, alcoholic precipitation was combined with centrifugation for a quarter of an hour at 13,000 × g after being left at 4 °C with the alcohol. Then, washing with acetone and EtOH (96%) and using a nylon membrane with 0.45-µm pore size (Whatman International) was done to obtain PF residues, which were gathered and allowed to dry at 23 °C in a desiccator [16] since PF yield% is equal to (We/Wf)×100, as Wf represents the dry weight of the macroalga. We represent the dry weight of extracted PF [17]. All characterization and applications were done in the Regional Center for Mycology and Biotechnology (RCMB).

### Characterization of the extracted fucoidan

#### PF sulfate content

The following process was used to determine the proportion of PF’s sulfate content, which is one of its key features. 0.6 mL agarose-BaCl_2_ reagent (0.02% and 0.5%) was added after 1.2 mL of 8% trichloroacetic acid had been added and thoroughly mixed with 1.1 mL of PF aqueous solution (0.05%), and when turbidity reaches its maximum, nearly after more than an hour, we shake the sample and measure optical density (OD) at 500 nm using a spectrophotometer [18].

#### Proximate analysis

The proximate analysis included the following: (i) elemental analysis C, H, N, and S via combustion (Vario MICRO cube, elementary, Germany), (ii) total sugars in trifluoroacetic acid (TFA) hydrolyzate was determined by the phenol sulfuric acid assay [19], which involved adding 2.5 ml of concentrated H_2_SO_4_ and 0.5 ml of 5% (v/v) phenol solution to 0.5 ml of hydrolyzed PF. (iii) soluble protein was determined using the Lowry method [20]. After shaking and 20 min of heating in a boiling water bath (BWB), the mixture was allowed to cool to room temperature (RT). The UV-VIS absorption of the solution at 490 nm was then measured using a spectrophotometer. The total amount of sugar was determined using a glucose standard curve (SC). (iv). The water content of the PF was determined as a % of the dry weight following a 24-hour igniting process at 103 °C in an oven [21]. (v)According to [22], 70 mg of dry PF samples were burned in a muffle furnace for 14 h at 550 °C to measure the ash concentration gravimetrically. (vi). The meta-hydroxy diphenyl technique was used to measure the quantity of uronic acid [23]. Using this approach, 2.4 ml of concentrated H_2_SO_4_ containing up to 120 mM sodium tetraborate was mixed with 0.4 ml of PF (1 mg/ml) in water. In a 6-ml tube that was marble-capped to keep condensation from contaminating the sample, PF was then heated in BWB for 20 min. The absorbance between 400 and 700 nm was measured after the tube was cooled and filled with up to 150µL of m-hydroxy diphenyl reagent, then kept at RT for 15 to 1 h, and uronic acid% was estimated via using an SC.

#### FTIR analysis

By using an FTIR spectrometer (Bruker, ALPHA, Germany) equipped with the attenuated total reflectance (ATR) method, 2.5 mg of PF powder was added to KBr (Merck^®^) pellets, which were used to record the transmission spectra. After deducting the atmospheric background interferences, with a resolution of 4.0 cm^− 1^, the spectra were acquired during 128 scans in the 4000–400 cm^− 1^ range [24].

#### SEM and EDX analysis

PF was examined using SEM (HRSEM; JSM-IT 200, Jeol, Japan) at a high vacuum and an acceleration voltage of 15 kV to gain a better illustration of the morphology and surface texture of PF. To prepare it for SEM investigation, the sample was physically vapor-deposited with gold (15 Å) for two minutes. The EDX spectrometer was used for elemental detection with the evaluation of their mass and atomic % in response to the X-rays that were released [24].

#### PF HPLC analysis

With minor adjustments, Toskas et al. description of acid hydrolysis was applied to a PF sample [25]. Thirteen milligrams of the material were combined with five milliliters of 4 M TFA in the reaction tube. For six hours, oven hydrolysis was carried out at 124 °C. The hydrolysate was evaporated at 40 °C. Following drying, 3 mL of DI H_2_O was used to dissolve the hydrolysate. The monosaccharide composition was ascertained using an HPLC (Agielnt, USA) apparatus that included a Binary HPLC pump with an injector, a refractive index detector (RI, 2410) at 35 °C, and a software monitor that was running the Breeze program. Supelco supplied the LC-NH2 column (SUPELCOILTM LC- NH2, 250 4.6 mm, 5 μm). 1.5 ml/min of an acetonitrile/water (85:15) solvent solution in the mobile phase. PF (10 µl) was added to the HPLC at a flow rate of 1.5 ml/min, using acetonitrile/water as the eluent. By comparing the chromatographic peaks with reference sugars (L-rhamnose, galactose, glucose, xylose, L-arabinose, fucose, and fructose) supplied by Sigma Aldrich, India, for monosaccharide analysis, the software application Breeze was able to identify the PF sugars.

#### PF thermal analysis

PF TGA was done using Netzsch DSC 204 with continuous nitrogen flushing to evaluate the highest PF saturation and melting point, by heating 1.71 mg PF from RT to 1000 °C with 20 °C per minute. The thermogram was obtained as the temperature increased [26].

#### Veridical of PF

The American Type Culture Collection provided the Vero cells (VCs), which were isolated from the kidney of an African green monkey (ATCC, Manassas, VA, USA). According to Vijayan et al. [27], VCs were cultivated twice a week in Dulbecco’s modified Eagle’s medium (DMEM) at RT in a wet environment with 5% CO2. Using the Spearman-Karber method for infectious virus counting by calculating the dose of infectious tissue culture (TCID50) [28], in confluent VCs, the cytopathogenic HAV HM175 strain of the hepatitis A virus was cultivated and examined. By the day PF was added for each well, VCs and HAV HM175 were planted in 96-well plates (WP) with a density of 2 × 10^5^ cells/100 L medium for cytotoxicity and antiviral test evaluation, respectively. Cells were grown as a control with or without DMSO and without PF at doses of 3000–2 µg/mL. OD at 590 nm was then measured with an ELISA reader (SunRise, TECAN, Inc., USA), using an MTT colorimetric test to estimate living cell % [29, 30]. The 50% cytotoxic concentration (CC50), or the dosage necessary to cause deadly effects in intact cells, can be found using the GraphPad Prism program with Acyclovir as a reference medication (San Diego, CA. USA), with the evaluation of maximum non-toxic concentration (MNT) was also ascertained.

The following equation could be used to assess the rate of viral inhibition:

$${\rm{(A - B)/(C - B) \times 100\% }}$$

Where A, B, and C represented PF OD with virus-infected cells, the virus control OD, and the cell control OD, respectively.

### Antimicrobial activity of PF

The antibacterial activity of PF was evaluated against *Pseudomonas aeruginosa* ATCC 9027, *Escherichia coli* ATCC8739, Methicillin-Resistant *Staphylococcus aureus* (MARSA) (ATCC 4330), and *Staphylococcus aureus* ATCC25923 using the agar well diffusion method, *Aspergillus fumigatus* (RCMB 002008), and *Candida lipolytica* (RCMB 005007 (1)). Pure colonies of the aforementioned strains must be cultivated overnight on an agar plate in order to prepare the inocula for the antimicrobial experiment. The bacterial strains must be inoculated into Mueller-Hinton broth, while the fungal strains must be inoculated into malt broth. After being prepared and autoclaved for sterilization, the Mueller Hinton Agar (MHA) medium was uniformly divided among Petri dishes (PD). Using a sterile glass spreader, 100µL of the pathogenic microbe culture broth (CFU 106 cells/mL) was carefully spread into different sterilized PD to produce a uniform layer of the bacterial suspension. After using a sterile cork borer to shape each plate into a well with a diameter of 6 mm, the plates were loaded with a DMSO solution of PF and the reference standard antibiotic, gentamicin (4 µg/ml). Sterile distilled water and gentamicin at concentrations of 4 µ g/mL were combined to create the gentamicin solution. PD was covered and maintained in an incubator at 37 °C. Well diameter (in mm) was measured when the wells displayed distinct inhibitory zones following 48 h at 28 °C for fungus and 24 h at 37 °C for bacteria. Three duplicates of the experiment were done to determine the mean ± SD.

#### MIC determination PF

Gentamicin and Ketoconazole (4 µg/ml), popular antibiotics, were utilized to estimate the MIC and MFC, respectively, using a microdilution technique [31]. Different PF and antibiotic concentrations were created using 5% DMSO. In Mueller Hinton broth (MHB) tubes, doses of 1.25, 2.5, and 5 mg/mL from PF with antibiotic (4 µg/ml) were mixed after 100 mL of the used strains (1.5 × 108 CFU/mL) were added. The MHB tubes used as controls were infected with the bacterium under investigation and incubated for a day at 37 °C. The lowest concentration of antibacterial and antifungal agents that prevented germs from developing observably was used to compute the MIC and MFC values [32].

### FRAP assay

Sutharsingh et al. [33] and Banerjee and Maulik [34] proposed methodologies for measuring PF’s reducing power. This method involves combining 1 milliliter of PF aqueous solution with 2.5 milliliters of 0.2 M sodium phosphate buffer (pH 6.6) and 2.5 milliliters of [K3Fe (CN)6] (1%, w/v) to reduce ferricyanide in the presence of varying PF concentrations. After 20 min at 50 °C, 2.5 ml of 10% w/v trichloroacetic acid was added for acidification, then centrifugation occurred at 1000 g for third an hour, finally OD was determined spectrophotometrically at 700 nm with respect to a blank (Milton Roy, Spectronic 1201), compared with ascorbic acid as a reference.

$$\eqalign{& Reducing\,capability\left( \% \right) \cr& = 100 - \left[ {{A_o} - {A_s}/{A_o} \times 100} \right] \cr} $$

Where A_0_ and A_s_ are absorbance control and PF, respectively [35].

### Antidiabetic activity

Using soluble starch as the substrate and Acarbose as a reference standard, the α-Amylase activity was estimated to determine the antidiabetic effect of PF. Using a pink-colored glucose oxidase assay, 20 µl amylase (1 g%) was mixed with 5 µl PF at concentrations (0–1000 µg/ml), and 100 µl phosphate buffer (pH = 6.9), then placed at RT for a quarter to hour. After incubation, add 20 µl of substrate-soluble starch (1 g %) and incubate for 20 min. Next, add 100 µl of glucose reagent and incubate for 20 min at room temperature. [36] and [37] reported that absorbance was measured at 490 nm.

## Result and discussion

### Fucoidan extraction yield and compositional analysis

**Fig. 1:**
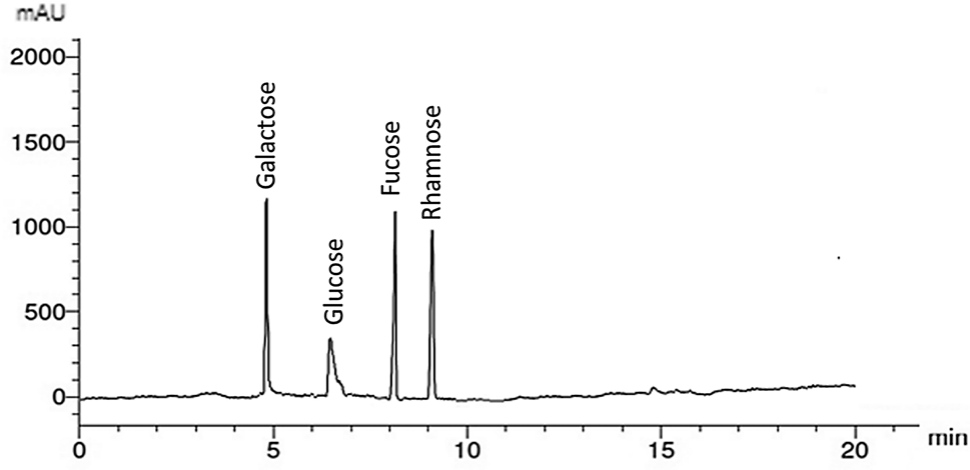
HPLC analysis of *Padina pavonica* fucoidan

The proximate analyses of PF are presented in Table 1. Twenty grams (dry weight) of *P. pavonica* powder yielded 3.56 g of PF, indicating that the yield of fucoidan extraction from *P. pavonica* was 17.8 ± 0.23%. This finding aligns with the estimates of [38], who reported the yield percentage of *P. pavonica* fucoidan to be between 5.65% and 40.76% DW. The PF sulfate content was recorded at 9.52 ± 0.19%, nearly identical to *Sargassum fluitans* (7.56%) [39]. Additionally, PF contains 12.95 ± 0.62% total sugars, 9.66 ± 0.42% water, 0.23 ± 0.02% protein, and 18.23 ± 0.15% ash, while the percentage of C, H, N, and S are 18.24 ± 0.25, 5.96 ± 0.29, 3.14 ± 0.55, and 10.32 ± 0.14, respectively. The HPLC study (Fig. 1) demonstrated that the PF compositions predominantly consist of glucose, rhamnose, fucose, and galactose (Table 1), with glucose being the minor component and galactose the major one. The sample exhibited a significant amount of fucose, which serves as a fingerprint for fucoidan; according to [40], *Padina* fucoidan was found to contain primarily fucose and galactose. *Sargassum tenerium* contained 39.04% fucose, and the water content of PF was 9.66 ± 0.42%, comparable to the uronic acid content of PF, which was 9.15 ± 0.87%. This roughly corresponds with the findings of [26], while the ash content of 18.23 ± 0.15% is in line with the Food Chemicals Codex (1981), which indicates that ash content can vary between 13% and 27% [41]. Reported that the H_2_O content in SP ranged from 10 to 15%. When compared to international standards, these findings conform to them.

**Table 1:** *Padina Pavonica* fucoidan extraction yield and compositional analysis

Parameter	Value (%) dry weight
PF yield	17.8 ± 0.23
Ash content	18.23 ± 0.15
Water content	9.66 ± 0.42
Protein content	0.23 ± 0.02
Sulphate content	9.52 ± 0.19
C content	18.24 ± 0.25
H content	5.96 ± 0.29
N content	3.14 ± 0.55
S content	10.32 ± 0.14
Total sugars	12.95 ± 0.62
Uronic acid	9.15 ± 0.87
**HPLC sugars (% dry weight)**	
Galactose content	37.45
Glucose content	2.05
Fucose content	34.45
Rhamnose content	26.05

#### FTIR

**Fig. 2:**
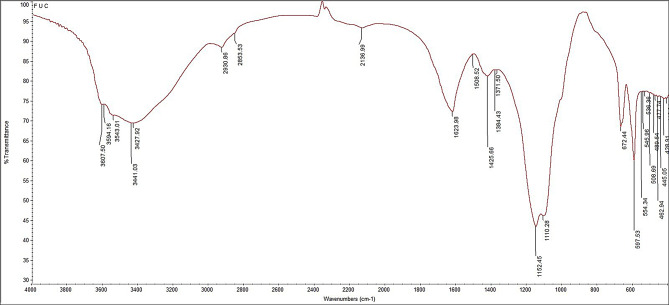
FT-IR analysis of *Padina pavonica* fucoidan

Figure 2 demonstrates the PF FT-IR analysis. The bands located between 3607.50 and 3427.92 cm^− 1^ were identified as having the carbohydrate O–H stretching tremor. Smaller bands and shoulders at 2930.86–2853.53 cm^− 1^ were attributed to CH in the pyranoid ring, and C-6 groups of fucose units were identified [42]. Found that the band at 2136.99 cm^− 1^ corresponds to the bending vibrations of alkyl groups (-CH2−, CH3), showing the presence of C = O stretching vibrations of O-acetyl groups. *P. pavonica*-derived fucoidan exhibited a shoulder at 1508.52, which is specific for amide-II [43]. The band at 1425.66 cm^− 1^ (galactose, xylose) was given the designation CH2. The distinctive absorption band of fucoidan at 1384.43 cm^− 1^ (S = O Stretching, Fucose, O-acetyl group) indicates the presence of sulfate groups. Absorbance at 1110.28 cm^− 1^ wavelength suggests vibration of C-O-SO3.

#### EDX and SEM of PF

**Fig. 3:**
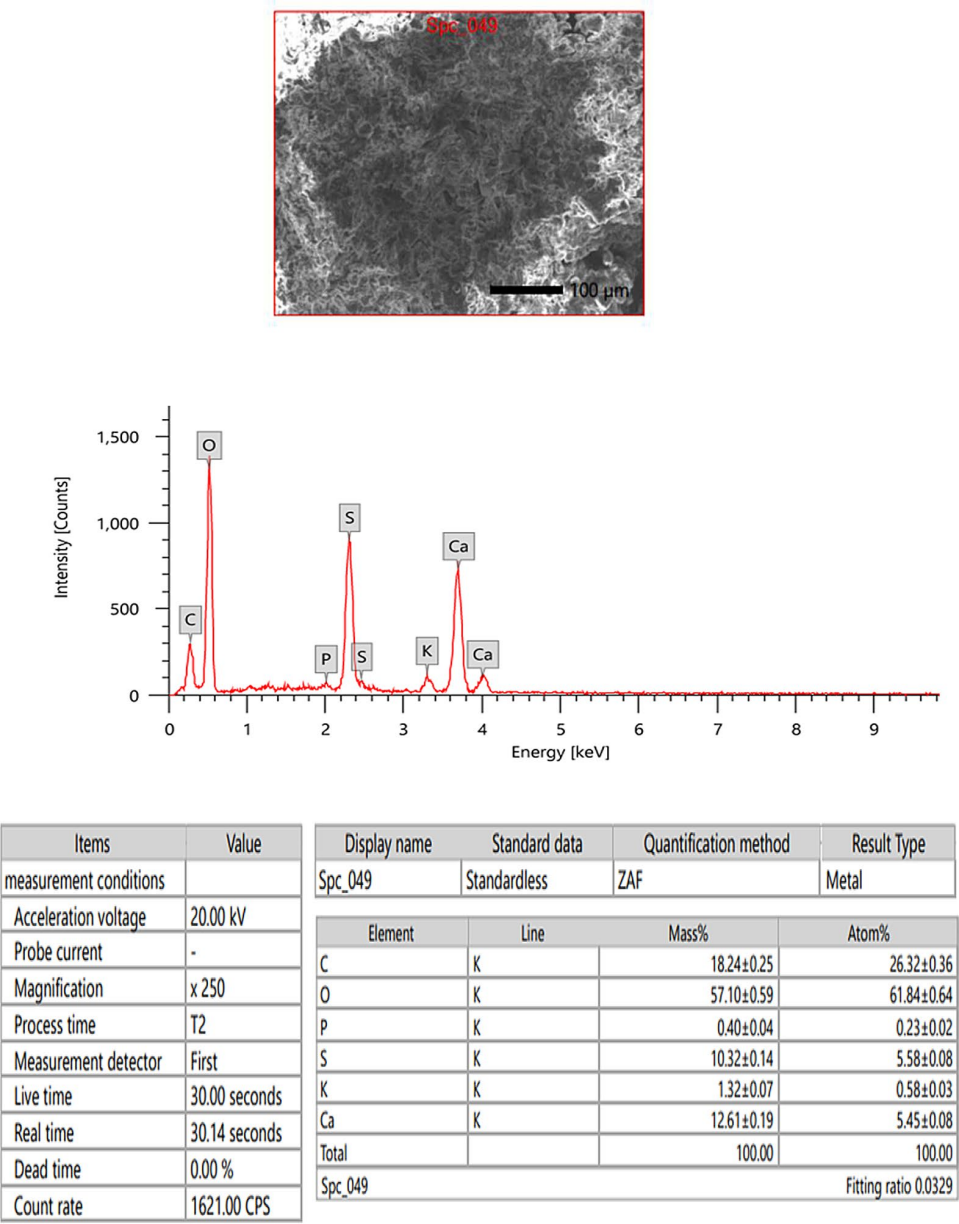
EDX analysis of *Padina pavonica* fucoidan

**Fig. 4:**
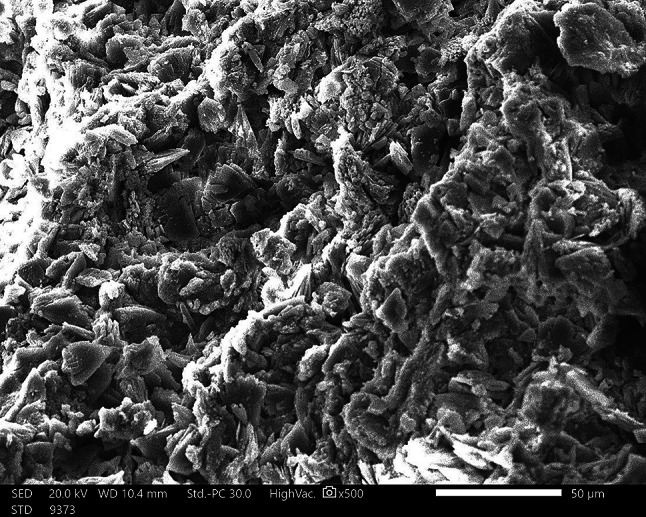
SEM image of *Padina pavonica* fucoidan using scale bar 50 μm

According to Fig. 3, PF contains O, C, S, K, Ca, and P with amounts 57.10 ± 0.59%, 18.24 ± 0.25%, 10.32 ± 0.14%, 1.32 ± 0.07%, and 12.61 ± 0.19%, respectively. Meanwhile, the extracted PF’s SEM micrographs confirmed a semi-crystalline and non-smooth texture (Fig. 4). The SEM image of PF displays an irregular shape similar to *Sargassum longifolium* [44] and a worm-like structure similar to *S. plagiophylum* [45].

#### TGA analysis of PF

**Fig. 5:**
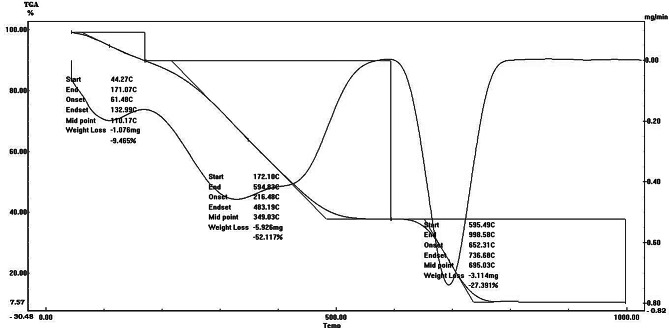
Thermogravimetric analysis of fucoidan from the brown seaweed *Padina pavonia*

The thermal stability of PF was assessed using the TGA (Fig. 5). The graphs show sample weight loss after continuous heating to 1000 °C. In the final stage (595.45–998.58 °C), the weight of PD changed to 3.114 mg with a weight loss percentage of 27.391, and the remainder represented the ash content, which might include polysaccharide minerals such as S, P, and CO_3_ [46]. The thermal breakdown began at 44.92 °C and reached 595.49 °C. These results agreed with [26] findings, which heated *Sargassum ilicifolium* fucoidan and discovered that the sample lost weight after being heated continuously to 800 °C. The thermal breakdown started at 41.92 °C, and the conclusions were reached at 580.47 °C. Due to their high carbohydrate content, all seaweeds, including *S. thunbergii*, *M. stellatus*, and *Ulva.* sp. showed a maximum weight loss between 170 and 280 °C [15].

### Biological activities of PF

#### Antiviral activity

**Fig. 6:**
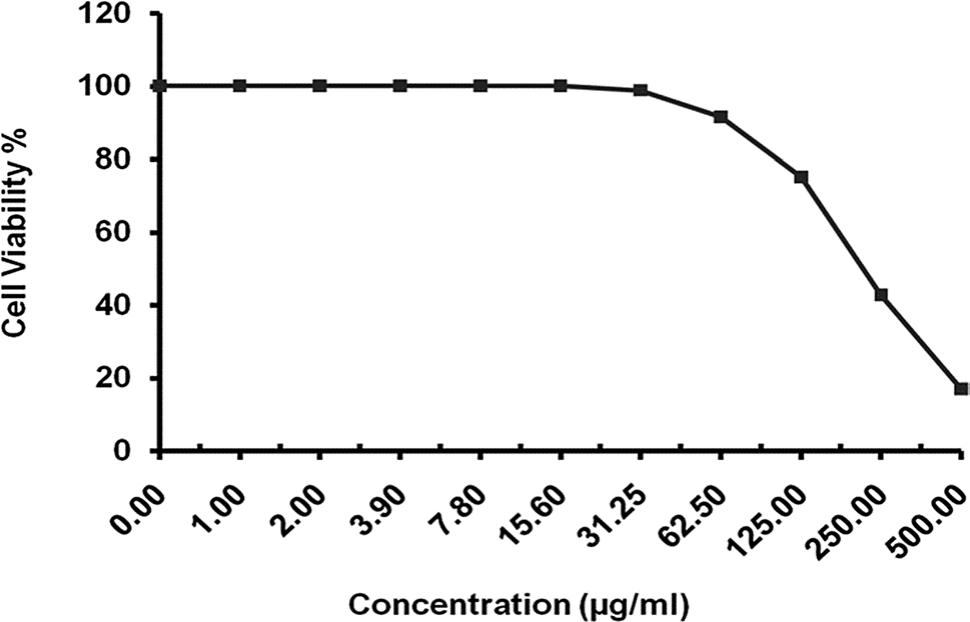
Cytotoxicity of *Padina pavonica* fucoidan upon normal Vero cell line

Concerning molecular weight and sulfated groups, PF exhibits promising antiviral efficacy [47, 48]. When PF is tested at an MNCC of 50 µg/mL, its antiviral activities on the herpes simplex type-1 virus (HSV-1) are shown in Table 2. With a 30.89% ± 0.84% inhibition, a CC50 of 210.42 ± 1.25 µg/mL, and an EC50 of 49.89 ± 0.88, PF demonstrated (++) moderate antiviral efficacy against HSV-1. The selectivity index ((SI) = 4.21), which shows the safety degree of PF, was calculated using the ratio of CC50—EC50 as it is a therapeutic index that showed that PF can be used as active antiviral medicines, as compounds with SI ≥ 2 are described as active [49]. The safety degree of PF on the Vero cell line was demonstrated in Fig. 6, where the viability percent remained 100% till 15.6 µg/ml, and this is very important information if PF is suggested for food or pharmaceutical applications. Algal sulfated polysaccharides have demonstrated their effectiveness, especially against pathogenic viruses in various stages of virus infection, according to [50]. Indeed, the unique structural characteristics of sulfated polysaccharides (SP) confer antiviral effects by obstructing a virus’s life cycle at various stages.

**Table 2:** The antiviral effects of *Padina Pavonica* fucoidan against herpes simplex type-1 virus (HSV-1) using acyclovir, reference drug when tested at maximum noncytotoxic Conc (MNCC)

Sample name	MNCC (µg/ml)	Antiviral effect on HSV-1 (%) Tested at MNCC	Antiviral effect on HSV-1 (Qualitative)*	Antiviral Efficiency		
				EC_50_	CC_50_	SI
*P. pavonica* fucoidan	50	30.89 ± 0.84	+++	49.89 ± 0.88	210.42 ± 1.25	4.21
Acyclovir Reference drug	20	88.30 ± 1.08	++++	4.07 ± 0.31	145.05± 6.93	35.63

Where (-): No antiviral activity (+): Weak antiviral activity (1-<25%) (++): Moderate antiviral activity (25-<50%) (+++): Good antiviral activity (50-<75%) (++++): Excellent antiviral activity (75–100%)

SP’s unique structural characteristics produce antiviral effects by obstructing viruses at different points of their life cycle. Sulfated polysaccharides can inhibit virus-host cell attachment and penetration, thus stopping the infection. Sulfated polysaccharides’ antiviral activity is achieved by blocking the virus’s interaction with the receptors by preventing it from adhering to them, thanks to their polyanionic characteristics, which enables them to deactivate viruses [51]. SP ought to attach to the virus’s surface amino acids [52]. Seaweed sulfated polysaccharides are preferable as a novel medicine due to their safety, antiviral activity, inexpensive manufacturing costs, and cytotoxicity [53]. *S. mcclurei* Fucoidan demonstrated similar anti-HIV effectiveness [54].

#### Antimicrobial activity

**Fig. 7:**
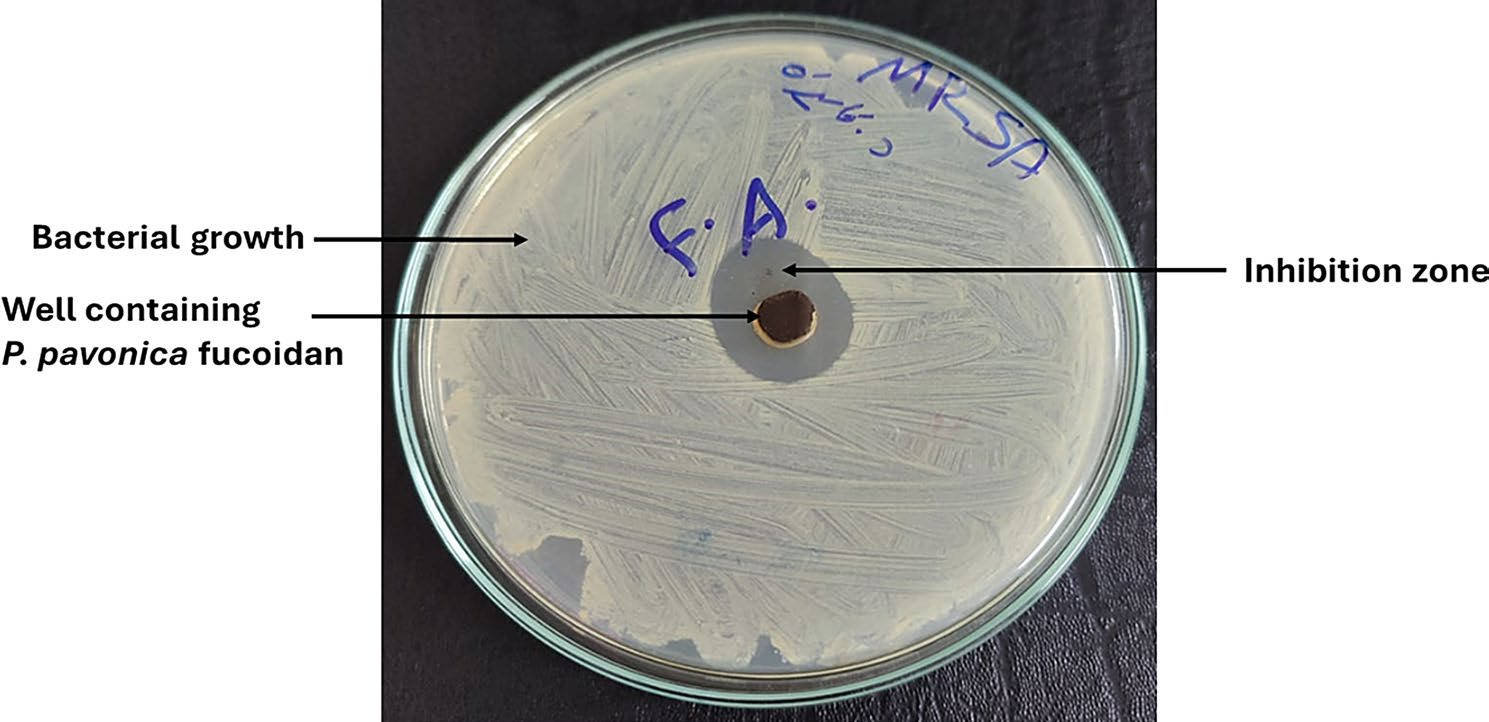
Antimicrobial activity of *Padina pavonica* fucoidan against MARSA

Figure 7 displays PF’s antimicrobial qualities against human microbiological pathogens. Table 3 displays the results. The maximum activity for MARSA was 18 ± 0.41 mm, which was greater than the reference medication gentamycin’s 15 ± 0.85 mm, and these findings agreed with [55, 56]. The results for the other infections were rather moderate, the MIC and MFC were estimated (Table 4) to be between 1.2 and 10 mg and 10 to 20 mg, respectively, and that was in agreement with [38]. The chemical structure and ester sulfate groups of polysaccharides are used to evaluate how well they kill microorganisms [57]. According to [58], different types of brown seaweed with high sulfate concentrations exhibit variable degrees of antibacterial activity.

**Table 3:** Antimicrobial activity of *Padina Pavonica* fucoidan against pathogenic bacterial and fungal strains (inhibition zone in mm)

Pathogenic strain	*P. pavonica* fucoidan	Reference drug
**Fungi**		**Ketoconazol**
*Aspergillus fumigatus* (RCMB 002008)	9	17
*Candida lipolytica* (RCMB 005007 (1))	11	18
**Gram positive bacteria**		**Gentamycin**
*Staphylococcus aureus* ATCC25923	15	24
*Methicillin-Resistant Staphylococcus aureus* (MARSA) (ATCC 4330)	18	15
**Gram negative bacteria**		**Gentamycin**
*Escherichia coli* ATCC8739	11	30
*Pseudomonas aeruginosa* ATCC 9027	17	27

**Table 4:** MIC of *Padina Pavonica* fucoidan against pathogenic strains

Pathogenic strain	*P. pavonica* fucoidan concentration mg/L
**Fungi**	
*Aspergillus fumigatus* (RCMB 002008)	20
*Candida lipolytica* (RCMB 005007(1))	10
**Gram positive bacteria**	
*Staphylococcus aureus* ATCC25923	2.5
*Methicillin-Resistant Staphylococcus aureus* (MARSA) (ATCC 4330)	1.25
**Gram negative bacteria**	
*Escherichia coli* ATCC8739	10
*Pseudomonas aeruginosa* ATCC 9027	1.25

#### Antioxidant activity

PF’s Antioxidant Activity Although PD’s antioxidant efficacy is not greater than that of vitamin C (the reference drug), Table 5 demonstrates that it is a potent antioxidant because the polysaccharide activated FRAP, and the activity (%) increased gradually, and these findings were supported by those of [59]. The maximum inhibition was attained at 1000 µg/mL with a percent of 81.82 ± 1.44, and the IC50 value of fucoidan was 113.21 ± 4.37 µg/mL. According to [60], the occurrence of reductants inhibiting peroxide generation via interacting with peroxide precursors is usually related to reducing properties. The findings indicate that PF’s promising reducing capability is what gives it its antioxidant action [61]. According to [62], the FRAP of fucoidan produced from *Padina tetrastromatica* increased dramatically with increasing fucoidan concentrations. Since the sulfate group in *Sargassum horneri*’s sulfated polysaccharide activates its reducing power [63].

**Table 5:** Antioxidant activity of *Padina Pavonica* fucoidan using FRAP assay

Sample concentration (µg/ml)	FRAP scavenging %	
	*P. pavonica* fucoidan	Ascorbic acid standard
0.5	0.84 ± 0.12	7.16 ± 1.31
1	1.93 ± 0.29	12.46 ± 1.56
2	3.18 ± 0.46	18.89 ± 1.46
3.9	4.59 ± 0.27	25.90 ± 1.21
7.8	7.41 ± 0.33	34.15 ± 1.43
15.6	11.23 ± 0.75	44.93 ± 1.15
31.25	19.76 ± 1.48	59.92 ± 1.61
62.5	31.94 ± 2.32	71.20 ± 1.88
125	54.20 ± 1.46	82.95 ± 1.09
250	63.85 ± 0.97	90.57 ± 1.17
500	70.49 ± 1.75	92.87 ± 0.21
1000	81.82 ± 1.44	94.19 ± 0.38
*IC*_*50*_	113.21 ± 4.37 µg/ml	20.89 ± 1.25 µg/ml

#### Antidiabetic activity

Table 6 demonstrates that PF inhibited α-amylase at all concentrations but did not exceed the efficacy of the reference drug Acarbose. The inhibition percentage of α-amylase is directly proportional to all tested concentrations, with 1000 µg/ml PF causing the highest inhibition percentage of 75.69 ± 1.05 and an IC50 of 117.56 ± 4.03 µg/ml. According to [64], fucoidan primarily reduces postprandial hyperglycemia, mitigates pancreatic β-cell damage, increases insulin production, and regulates glucose metabolism to lower blood glucose levels. Postprandial hyperglycemia is a feature of type 2 diabetes caused by the rapid breakdown of starch into glucose, which raises blood glucose levels [65]. α-amylase breaks down dietary starch into a significant amount of maltose, which is then converted into glucose by α-D-glucosidase [66]. Thus, blocking α-amylase activity helps regulate blood glucose levels [67, 68] found that α-amylase had two inhibitory actions that lower hyperglycemia in diabetics. Research suggests that when fucoidan levels rise, its inhibitory effects on α-amylase also increase [69–71] discovered that fucoidan’s hydroxyl group creates hydrogen bonds with α-amylase, inhibiting the enzyme. Fucoidan’s oxygen ion interacts with α-amylase through a strong hydrogen bond.

**Table 6:** Antidiabetic activity of *Padina Pavonica* fucoidan using α- amylase enzyme

Sample concentration (µg/ml)	α- Amylase Enzyme Inhibition (%)	
	*P. pavonica* fucoidan	Acarbose reference drug
0.5	2.98 ± 0.18	18.42 ± 0.68
1	6.45 ± 0.47	24.75 ± 1.39
2	9.83 ± 0.29	30.97 ± 0.65
3.9	14.95 ± 0.68	38.54 ± 0.28
7.8	20.83 ± 0.71	45.03 ± 0.82
15.6	27.04 ± 0.62	50.78 ± 0.75
31.25	33.21 ± 1.03	57.23 ± 0.91
62.5	40.67 ± 1.79	68.91 ± 0.32
125	51.26 ± 1.32	76.86 ± 0.44
250	58.28 ± 0.64	89.26 ± 0.82
500	64.91 ± 2.37	94.91 ± 0.73
1000	75.69 ± 1.05	96.73 ± 0.59
*IC*_*50*_	117.56 ± 4.03 µg/ml	14.54 ± 0.86 µg/ml

## Conclusion

It is consequently assumed that *Padina pavonica* is an excellent producer of fucoidan. The examination of isolated PF revealed the presence of promising sulfate content, which enhances its biological activities, particularly its anti-diabetic properties. The fucose content further establishes PF as an effective natural product with antioxidative, antiviral, and antimicrobial effects. Furthermore, fucoidan derived from *Padina pavonica* of Rocky Bay in Abu Qir, Alexandria, contains a significant number of mineral components, as demonstrated by EDX analysis, indicating its potential as a powerful constituent in drug and food manufacturing. While high concentrations of fucoidan caused toxicity in normal Vero cell lines, we tested them to study their biological effects; as such, high concentrations can be used as a disinfectant to kill microorganisms. We also recommend advanced studies to mitigate the toxic effects of these high concentrations to harness their strong biological activities.

## Data Availability

The manuscript contains all relevant data and materials.
